# Supplementary material for: The Preventive Effects of Salubrinal against Pyrethroid-Induced Disruption of Adult Hippocampal Neurogenesis in Mice
Source: Int J Mol Sci. 2023 Oct 26;24(21):15614. doi: 10.3390/ijms242115614 (PMC10648946; doi:10.3390/ijms242115614)
Supplement: Supplementary file 1 [file ijms-24-15614-s001.zip › ijms-2641676-supplementary.pdf]

Table S1. Total BrdU+, Ki-67+, DCX+, CHOP+, and caspase-12+ cells in the DG of the hippocampus following acute deltamethrin exposure in mice.

**Cell Type**	**Control**	**Salubrinal**	**Deltamethrin**	**Salubrinal + Deltamethrin**
BrdU+ cells	183	177	81*	150#
Ki-67+ cells	245	240	147*	218#
DCX+ cells	1130	1147	775*	1017#
CHOP+/BrdU+ cells	15	13	56*	22#
CHOP+/Ki67+ cells	14	12	60*	24#
Casp 12+/BrdU+ cells	15	12	66*	30#
Casp 12+/Ki67+ cells	16	15	69*	27#

Total BrdU+, Ki-67+, DCX+, and CHOP+ cells were visualized through immunofluorescence. Positive cells in the subgranular zone (SGZ) for BrdU and Ki-67, and in the SGZ and granule cell layer (GCL) for DCX, were manually counted at high magnification (40×) using an All-in-One Fluorescence Microscope (BZ-X810, Keyence Corporation, Itasca, IL). Positive cell quantification was performed on 12 sections per animal with 5 animals in each group. The group mean was obtained by averaging the total counts from these sections. Statistical comparisons were made using two-way ANOVA with Bonferroni's post hoc test. An asterisk (*) indicates a significant difference from the control group and a hashtag (#) indicates a significant difference from deltamethrin when compared with the deltamethrin + salubrinal group (BrdU+ cells: F 3,12 = 7.29; $p < 0.048$; Ki-67+ cells: F3,16 = 3.77; $p < 0.032$; DCX+ cells: F 3,16 = 3.67; $p < 0.035$; CHOP+/BrdU+: F 3,12 = 16.85; $p < 0.001$; CHOP+/Ki-67+ cells: F 3,12 = 11.99; $p < 0.005$; Caspase 12+/BrdU+ cells: F 3,12 = 10.20; $p < 0.001$; Caspase 12+/Ki67+ cells: F 3,12 = 34.96; $p < 0.001$).

Table S2. Total BrdU+, Ki-67+, DCX+ and CHOP+ cells in the DG of the hippocampus of caspase-12 KO mice following acute exposure to deltamethrin.

**Cell Type**	**WT Cont**	**Caspase 12 KO Cont**	**WT Del**	**Caspase 12 KO + Del**
BrdU+ cells	171	167	93*	153#
Ki-67+ cells	255	287	112*	206#
DCX+ cells	1209	1231	486*	891#
CHOP+/BrdU+ cells	12	9	56*	22#
CHOP+/Ki67+ cells	11	10	62*	30#

Total BrdU+, Ki-67+, DCX+, and CHOP+ cells were visualized through immunofluorescence. Positive cells in the subgranular zone (SGZ) for BrdU and Ki-67, and in the SGZ and granule cell layer (GCL) for DCX, were manually counted at high magnification (40×) using an All-in-One Fluorescence Microscope (BZ-X810, Keyence Corporation, Itasca, IL). Positive cell quantification was performed on 12 sections per animal with 5 animals in each group. The group mean was obtained by averaging the total counts from these sections. Statistical comparisons were made using two-way ANOVA with Bonferroni's post hoc test. An asterisk (*) indicates a significant difference from the control group and a hashtag (#) indicates a significant difference from deltamethrin when compared with the deltamethrin + salubrinal group (BrdU+ cells: F 3,12 = 8.81; $p < 0.0023$; Ki-67+ cells: F 3,16 = 4.92; $p < 0.01$; DCX+ cells: F 3,16 = 8.13; $p < 0.002$; CHOP+/BrdU+ cells: F 3,12 = 18.21; $p < 0.001$; CHOP+/Ki67+ cells: F 3,12 = 16.01; $p < 0.002$)
